# Identification of replicative aging and inflammatory aging signatures via whole-genome CRISPRi screens

**DOI:** 10.1186/s13059-025-03683-7

**Published:** 2025-08-06

**Authors:** Lingling Wu, Xiang Zhu, Yanxia Liu, Dehua Zhao, Betty Chentzu Yu, Zheng Wei, Xueqiu Lin, Lei S. Qi

**Affiliations:** 1 Department of Bioengineering, Stanford University, Stanford, CA 94305 USA; 2 Computational Biology Program, Fred Hutchinson Cancer Center, Seattle, WA 19024 USA; 3 Translational Data Science IRC, Fred Hutchinson Cancer Center, Seattle, WA 19024 USA; 4 Department of Statistics, The Pennsylvania State University, University Park, PA 16802 USA; 5 Huck Institutes of the Life Sciences, The Pennsylvania State University, University Park, PA 16802 USA; 6 Department of Statistics, Stanford University, Stanford, CA 94305 USA; 7 Sarafan ChEM-H, Stanford University, Stanford, CA 94305 USA; 8 Chan Zuckerberg Biohub - San Francisco, San Francisco, CA 94158 USA; 9 Current Address: Calico Life Sciences, South San Francisco, San Francisco, CA 94080 USA; 10 Currrent Address: Epicrispr Biotechnologies, South San Francisco, San Francisco, CA USA; 11 Currrent Address: Genomic Sciences, GSK, San Francisco, CA USA

## Abstract

**Background:**

Aging is a major risk factor for chronic diseases and cancer. Cellular aging, particularly in adult stem cells, offers a high-throughput framework for dissecting the molecular mechanisms of aging.

**Results:**

We perform multiple genome-wide CRISPR interference (CRISPRi) screenings in human primary mesenchymal stem cells derived from adipose tissue during either replicative senescence or inflammation-induced senescence. These screens reveal distinct sets of potential novel regulators specific to each senescence pathway. Combining our perturbation-based functional genomic data with 405 genome-wide association study datasets, including 50 aging-related studies, we find that the inflammatory aging signatures identified from CRISPRi screenings are significantly associated with diverse aging processes, suggesting novel molecular signatures for analyzing and predicting aging status and aging-related disease.

**Conclusions:**

The signatures verified through comprehensive functional genomics and genetic analyses may provide new targets for modulating the aging process and enhancing the quality of cell therapy products.

**Supplementary Information:**

The online version contains supplementary material available at 10.1186/s13059-025-03683-7.

## Background

Aging is an inevitable process with the gradual loss of tissue and organ stability. It is a major risk factor for cancer and chronic diseases, such as neurodegenerative, metabolic, and cardiovascular diseases [1]. Aging and cancer are closely linked, with significant overlap in their underlying molecular pathways [2]. A hallmark of aging is cellular senescence, a fundamental mechanism in which cells permanently lose their replicative capacity [3]. Systematic dissection of the genetic drivers of replicative senescence is critical to interrogating novel mechanisms of aging.

In parallel, inflammaging, chronic, sterile, low-grade inflammation, arises with the accumulation of senescent cells and advancing age, plays a major role in the development of aging-related diseases, including tumors [4, 5]. However, to date, the identification of senescence factors has focused primarily on replicative senescence [6–10]. A systematic comparison of genes involved in both replicative senescence and inflammaging is needed to elucidate distinct and shared aging pathways, and to facilitate the identification of therapeutic targets for age-related diseases, including cancer.

Mesenchymal stem cells (MSCs) are a multipotent adult stem cell population widely distributed across different adult tissues [11]. Importantly, MSCs possess the ability for self-renewal and differentiation into mesodermal lineages, as well as non-mesodermal cell types such as myocytes, cardiomyocytes, and hepatocytes, making them a promising tool for cellular therapy. However, the inflammaging milieu in elderly individuals reduces the number and function of MSCs, resulting in the cellular senescence of MSCs characterized by reduced self-renewal and angiogenesis of MSCs [12, 13]. Senescent MSCs have been shown to facilitate tumor growth in a variety of cancer contexts [14, 15]. Therefore, a comprehensive understanding of MSC senescence—by comparing replicative arrest and inflammaging—will not only reveal novel aging pathways but also aid in the development of more effective cellular therapies for aging-related diseases, including cancer.

To study the mechanisms of MSC senescence, we performed multiple genome-wide CRISPR interference (CRISPRi) transcriptional repression screenings in human primary adipose-derived MSC. We identified potential novel factors in modulating MSC senescence in the context of both replicative senescence and inflammatory-induced senescence. Combining our perturbation-based functional genomic data with 405 GWAS datasets from 50 aging-related studies, we demonstrated that the inflammatory aging signatures identified from CRISPRi screens are significantly associated with aging processes across diverse organ systems. CRISPRi screening combined with comprehensive bioinformatic analysis suggested novel molecular signatures indicative of aging status and aging-related disease, while also highlighting novel targets to modulate aging processes and improve MSC-based cellular therapies.

## Results

### Genome-wide CRISPRi screening reveals novel senescence regulators in human primary adipose-derived MSC

Replicative senescence is a process during which somatic cells cultured in vitro enter an irreversible cell cycle arrest state. This process has been used as a model system to study the molecular mechanisms underlying cellular senescence. To establish an in vitro screening assay using MSCs, we first validated that replicative senescence could be recapitulated by growing primary MSCs to their replicative limit. We observed increases in the number of β-Gal positive cells, a widely used senescent marker, and decreases in proliferation rates (Additional file 1: Fig. S1A–C). Additionally, we observed elevated p21 and p16 mRNA expression in late-passage MSCs compared to early-passage MSCs (Additional file 1: Fig. S1D–E), which are molecular biomarkers for cellular senescence [1].

**Fig. 1:**
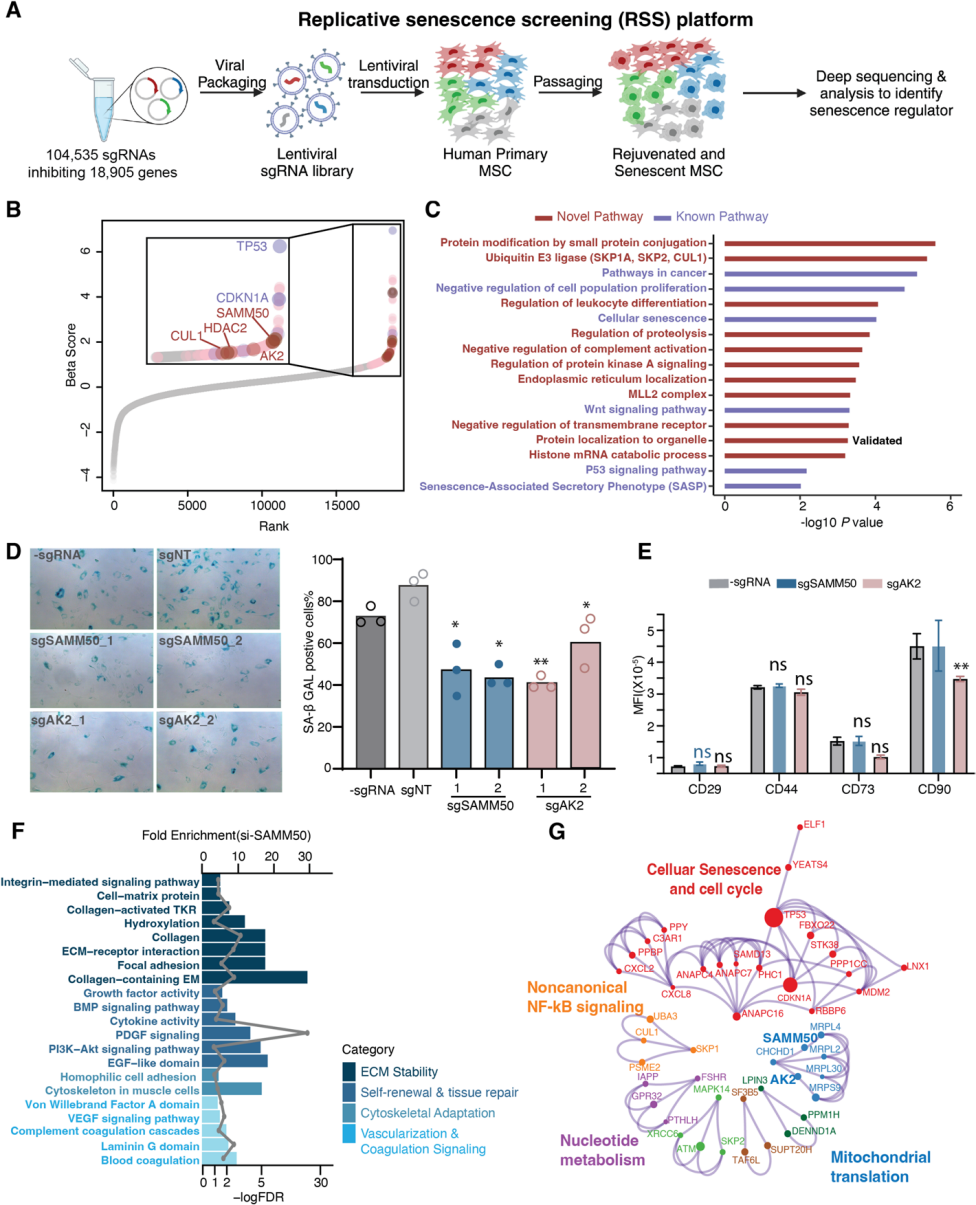
Genome-wide CRISPRi-based screen for human replicative senescence-promoting genes. **A** Schematic outlining the design of the CRISPRi-based screen to identify senescence-promoting genes in the primary human MSCs. Low MOI sgRNA library was transduced into primary human MSCs. After puromycin selection, dCas9 were transduced into the MSCs with sgRNA expression. The rejuvenated MSCs were deeply sequenced at approximately 8 weeks. Figure were created with BioRender.com. **B** Enrichment score calculated for each gene as beta rank. Genes detected in the screens are shown as dots in the diagram; the purple dots indicate well-known pro-aging genes in the screen; the dark red dots indicate novel findings. **C** Representative GO terms and pathways enriched in the candidate genes from the screen. The results were analyzed by Metascape. The purple bars indicate well-known pro-aging pathways; the dark red bars indicate novel findings from this screen. **D** SA-β-gal staining of human primary MSCs upon perturbation of two novel pro-aging genes (*SAMM50* and *AK2*). *n* = 3 biological replicates. Left panel: the microscope image of senescent cells showed as blue. Right panel: bar plots of the percentage of positive SA-β-gal cells. **E** Expression of 4 different biomarkers for MSCs upon perturbation of *SAMM50* and *AK2*. *n* = 2 biological replicates with 3 technical replicates. **F** Gene ontology analysis of DEGs upon inhibition of *SAMM50* in MSCs from DAVID functional enrichment analysis. Bar plots represent the FDR value and fold enrichment for GO terms. **G** Metascape visualization of the protein–protein interaction network of the enriched genes in the screen was applied. The colored points indicated beta value. (*p < 0.05, **p < 0.01,ns non-significant)

To identify novel regulators of replicative senescence in MSCs, we conducted a CRISPRi screen on the genome-wide scale [16–18] to systematically evaluate whether individual genes promoted cellular senescence. We generated a lentiviral library, encoding 104,535 single guide RNAs (sgRNAs) targeting 18,905 genes (approximately 5 sgRNAs per gene). The library also contained 1895 non-targeting sgRNAs, serving as important quality controls for true hits of the screen. We transduced the lentiviral sgRNA library into primary MSCs stably expressing dCas9-KRAB with a low multiplicity of infection (Methods) and allowed the sgRNA-incorporated MSCs to proliferate in the culture. The cells exhibited significant growth arrest after approximately 20 generations. Then, the initial and final cell populations were collected and sequenced to characterize the sgRNA presentation in each population. We termed this genome-wide CRISPRi screening platform as replicative senescence screening (RSS) (Fig. 1A).

We calculated the beta score of each gene by comparing the relative abundance of sgRNAs targeting given genes between the initial and late passage populations (Fig. 1B, Additional file 1: Fig. S2A and B, Additional file 2: Table S1). The beta score was used to quantitatively measure the gene essentialities: a positive beta score means a gene is enriched and this gene functions positively in cell senescence; and a negative beta score means a gene is depleted and the gene is an essential gene in cell fitness or functions negatively in cell senescence. To validate the predictive performance of the RSS platform, we conducted receiver operating characteristic (ROC) analysis using predefined gold-standard essential genes (KEGG_Ribosome, PMID: 33010154), non-essential genes (PMID: 27111720), and well-characterized pro-senescence genes (Additional file 3: Table S2). This approach allowed us to assess whether our screening datasets are robust and biologically meaningful (Additional file 1: Fig. S2C). The area under the curve (AUC) values exceeded 0.7 with significant *P*-values (< 0.01), demonstrating that our RSS screening is a biologically solid platform to identify essential genes and pro-senescence genes.

Supporting the validity of the screen, pro-senescence genes, such as *TP53* and *CDKN1A*, were significantly enriched (Fig. 1B). These two genes are major components of the MDM2/p53 axis, whose dysfunction is well known to be linked to premature aging [19]. Additionally, pathway enrichment analysis also highlighted reported pathways related to cellular senescence, including genes in the p53 signaling pathway, the senescence-associated secretory phenotype (SASP) pathway, and pathways related to cellular fitness and cancer (Fig. 1C, Additional file 4: Table S3). Altogether, these results confirmed the RSS platform provides a systematical way to identify genes and pathways related to replicative senescence.

The novel genes identified by the RSS platform are a comprehensive resource to pinpoint new regulators for rejuvenating MSC. Interestingly, genes like TP53 in primary MSCs not only affected cellular senescence (Additional file 1: Fig. S2D and S2E) but also significantly altered the expression of identity markers on MSCs including CD29, CD44, CD73, and CD90 (Additional file 1: Fig. S2F), suggesting these genes are not suitable targets for rejuvenating MSCs. Thus, it is critical to identify novel regulators for cellular senescence without changing the properties of MSCs. From our screening, besides the classical MDM2/p53 axis, we observed the enrichment of novel pathways, including protein localized in subcellular organelles, regulators of proteolysis, ubiquitin E3 ligase, and genes involving the protein modification process by small protein conjugation (Fig. 1C).

Previous studies have categorized the localization of age-altered proteins in yeast and found that homologs of some of our screened hits were particularly localized in the mitochondria [20]. Mitochondrial dysfunction is closely associated with cellular senescence, although the underlying mechanisms remain incomplete [21]. We validated top-ranked senescent regulators from the “protein localization to organelle” pathway, such as SAMM50 and AK2, which are two important proteins functioning in the mitochondrial membrane. Notably, CRISPRi-mediated inhibition of either of them rejuvenated MSCs (validated by two different sgRNAs for each gene to rule out potential off-target effects), as shown by reduced SA-β GAL staining (Fig. 1D). Importantly, perturbation of these two genes, particularly SAMM50, did not affect the expression of identity markers on MSCs including CD29, CD44, CD73, and CD90 (Fig. 1E).

To further investigate the underlying mechanism of mitochondrial membrane proteins as senescence regulators, we performed RNA-seq and measured whole genome transcriptomic changes caused by SAMM50 inhibition in primary MSCs (Additional file 1: Fig. S3A). The differentially expressed genes (DEGs) caused by *SAMM50* repression enriched in pathways related to self-renewal, tissue repair, and cytoskeletal adaptation (Fig. 1F), which is consistent with the phenotype of rejuvenated MSCs (Fig. 1D). Furthermore, extracellular matrix (ECM) related pathways, also enriched in DEG analysis, including collagen and extracellular structure organization, are consistent with the critical role of ECM in MSC function. Particularly, young ECM environments have been shown to “rejuvenate” aged MSCs via modulating their extracellular environment [22]. Thus, our results suggested that SAMM50 is a potent pro-aging factor that promotes human stem cell senescence through disruption of ECM integrity, potentially contributing to fibrosis and senescence-associated tissue deterioration.

To draw a functional network and view these candidates in protein–protein interaction modules, we utilized MCODE networks to identify novel modules [23]. We observed several intriguing modules heavily involved in the replicative senescence process, including mitochondrial translation, FoxO signaling, GPCR downstream signaling, noncanonical NF-kB signaling, and nucleotide metabolism (Fig. 1H). Notably, NF-kB signaling is a critical downstream pathway of multiple responses, such as reactive oxygen species, TNFα, IL-1β, and LPS, suggesting a link between aging and chronic inflammation. This observation is consistent with the observation that aging often happens along with chronic inflammation in aged humans. Thus, a platform that mimics the natural aging process under chronic inflammation is required to recapitulate the aging process in vivo and study senescent regulators specifically involved in inflammaging.

### Genome-wide CRISPRi screening reveals novel senescence regulators in inflammaging

Inflammaging is a chronic pro-inflammatory state and a pervasive hallmark of aging [24]. It is known that senescent cells secrete multiple inflammatory factors, chemokines, and matrix proteases, known as SASP. SASP from accumulated senescent cells in aged humans promotes inflammaging [25], creating a pro-inflammatory environment, which in turn, further induces cellular senescence and propagates it to adjacent cells [26]. As an important component of SASP, IL-6 is a prominent inflammatory cytokine linked to inflammaging and potentially many age-related diseases [27, 28]. Particularly, IL-6 can enhance cell senescence through autocrine and paracrine pathways [29]. Furthermore, research has linked IL-6 to the senescence of bone mesenchymal stem cells in a high-fat diet-induced bone loss model [30]. Besides, IL-6 contributes to tumorigenesis by promoting growth, metastasis, and therapy resistance through modulation of the tumor microenvironment [31, 32].

**Fig. 2:**
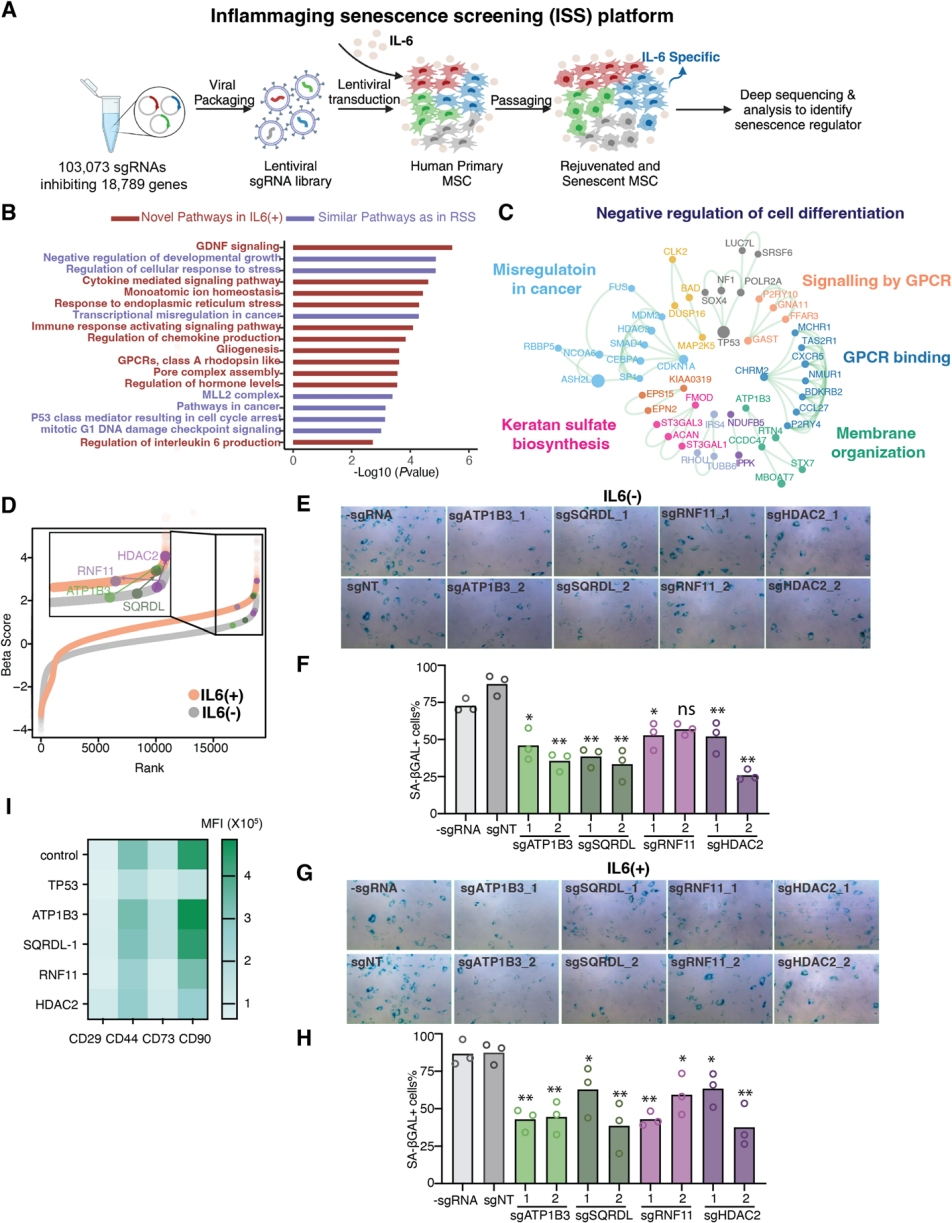
A modified genome-wide CRISPRi-based screen for human inflammatory senescence-promoting genes. **A** Schematic outlining the design of a CRISPRi-based screen to identify inflammatory senescence-promoting genes in the primary human MSCs. Low MOI sgRNA library was transduced into primary human MSCs. After puromycin selection, dCas9 were transduced into the MSCs with sgRNA expression. The rejuvenated MSCs incubated with IL-6 (10 ng/ml) were deeply sequenced at approximately 8 weeks. Figures were created with BioRender.com. **B** Representative GO terms and pathways enriched in the candidate genes in the screen. The purple bars indicate similar pro-aging pathways found in both RSS and ISS screens; the dark red bars indicate novel pathways identified by this ISS screen. **C** Metascape visualization of the genetic interaction network of the enriched genes in the screen was applied. The colored points indicate densely connected network components identified by the MCODE algorithm. **D** Scatter plots of beta scores calculated from two screen platforms. **E**–**H** SA-β-gal staining of human primary MSCs upon perturbation of four novel pro-aging genes (*ATP1B3*, *SQRDL*, *RNF11*, and *HDAC2*) without IL-6 (**E** and **F**) and with IL-6 (**G** and **H**). **E** and **G** Light microscopic images of cells. Blue dots mean the positive staining. **F** and **H** Quantification of SA-β-gal positive staining images by ImageJ software. *n* = 3 biological replicates. Each gene was tested by 2 different sgRNAs. **I** Expression of 4 different biomarkers for MSCs upon perturbation of four novel pro-aging genes (*ATP1B3*, *SQRDL*, *RNF11*, and *HDAC2*). *n* = 2 biological replicates with 3 technical replicates. (*p < 0.05, **p < 0.01, ns non-significant)

To systematically study the senescence regulators in inflammaging, we further developed a CRISPRi-based inflammatory senescence screening (ISS) system. First, we confirmed that, as expected, IL-6 significantly increased the number of senescent cells (β-Gal positive) in early-passage MSCs (Additional file 1: Fig. S4A). To assess the optimal concentration of IL-6, we quantified p16 and p21 expression across various IL-6 concentrations in early passages and determined the optimal concentration of 10 ng/ml (Additional file 1: Fig. S4B). Compared to this low IL-6 concentration, a significantly high IL-6 concentration was observed to recapitulate acute infections in vitro. For instance, the IL-6 concentration in peripheral blood mononuclear cells (PBMCs) stimulated by a component of Gram-negative bacteria (lipopolysaccharides) was approximately 100 ng/ml [33]. The mild concentration causing inflammatory senescence was consistent with the phenomenon whereby aging-associated elevation of IL-6 is usually mild. Particularly, the IL-6 levels observed in acute infections (e.g., COVID-19 patients) could reach approximately 70 pg/ml [34], while the aging-associated elevation of IL-6 is mild (e.g., ~ 3.5 pg/ml for men and ~ 2.1 pg/ml for women aged ≥ 85 years old) [35]. Therefore, we chose to add the low concentration of 10 ng/ml IL-6 to mimic inflammaging to investigate the senescence regulators that specifically function in inflammaging (Fig. 2A). Similar to the RSS platform, the ISS platform demonstrated robustness, as evidenced by consistent biological replications (Additional file 1: Fig. S4C and D) and strong performance in ROC analysis in identifying essential genes and pro-senescence genes (Additional file 1: Fig. S4E).

We conducted the ISS screening with 10 ng/ml IL-6 and identified senescence regulators enriched in pathways shared with the RSS screen, as well as novel pathways related to inflammation (Fig. 2B, Additional file 4: Table S3). MCODE network analysis identified novel pathways potentially involved in senescence, including the MLL4 complex, mRNA metabolic processes, membrane organization, keratan sulfate/keratin metabolism, and membrane trafficking, highlighting new areas for aging study [23] (Fig. 2C). Notably, IL-6 treatment enhanced the selective pressure, leading to more pronounced enrichment scores in both directions, and thus expanded the dynamic range of the ISS screen (Fig. 2D).

To further validate our findings, we selected four genes (*ATP1B3*, *SQRDL*, *RNF11*, and *HDAC2*) identified from both the RSS and ISS platforms. Among these, *ATP1B3* and *RNF11* are involved in membrane organization, *HDAC2* functions in epigenetic regulation, and *SQRDL* is associated with mitochondrial pathways. Consistent with earlier observations, the enrichment scores for these four genes were more significant in the ISS screen than in the RSS screen. Functional validation demonstrated that inhibition of these genes significantly reduced the number of SA-β GAL positive cells in MSC cultures, both with and without IL-6 treatment (Fig. 2E–H). Of note, compared to *TP53*, inhibition of these four genes showed a much smaller effect on the expression of MSC identity markers (Fig. 2I). These results suggested that the addition of IL-6 enhances the ability of CRISPRi screening for identifying senescence regulators.

### Classification of senescence genes identifies inflammaging-specific senescence genes

**Fig. 3:**
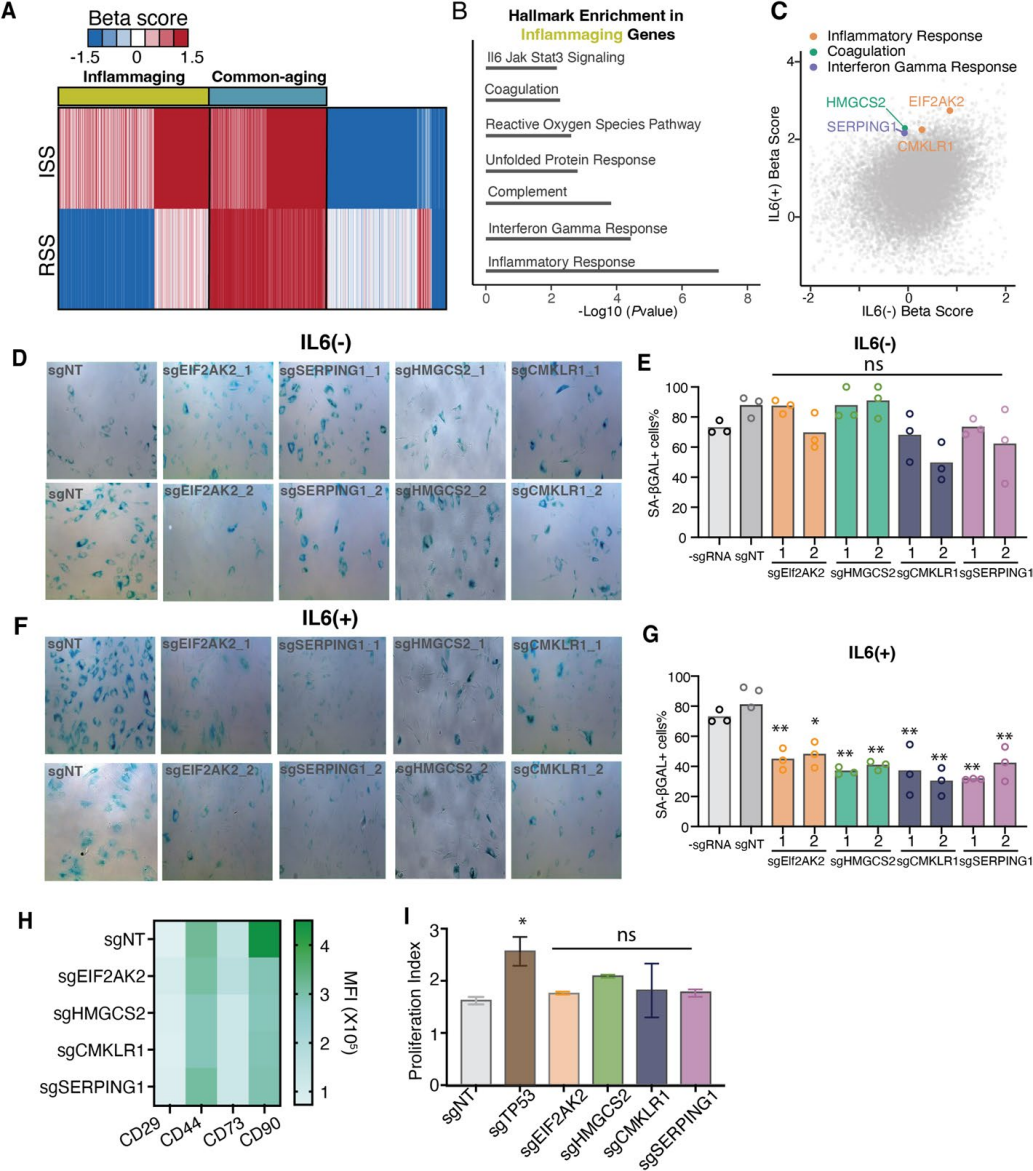
Identification of inflammatory-specific senescence genes. **A** Heatmap of K-means clusters of significantly enriched and deleted genes in both RSS and ISS platforms. **B** Representative Hallmark genes enriched in the inflammaging genes. **C** Scatter plot showing the enrichment score of genes from two screening platforms. Each dot presents an individual gene from two screening platforms. Colored dots represent the four inflammaging genes being validated later which belong to different pathways. **D**–**G** SA-β-gal staining of human primary MSC perturbation upon four inflammaging genes (*EIF2AK2*, *SERPING1*, *HMGCS2*, and *CMKLR1*) without IL-6 (**D** and **E**) and with IL-6 (**F** and **G**). **D** and **F** Light microscopic images of cells. Blue dots mean the positive staining. **E** and **G** Quantification of SA-β-gal positive staining images by ImageJ software. *n* = 3 biological replicates. Each gene was tested by 2 different sgRNAs. (H) Expression of 4 different biomarkers for MSCs upon the perturbation of four inflammaging genes (*EIF2AK2*, *SERPING1*, *HMGCS2*, and *CMKLR1*). *n* = 2 biological replicates (**I**) proliferation index of MSCs after perturbation of four inflammaging genes (*EIF2AK2*, *SERPING1*, *HMGCS2*, and *CMKLR1*). The proliferation index was calculated by FlowJo software. *n* = 2 biological replicates with 3 technical replicates. (*p < 0.05, **p < 0.01, ns non-significant)

When gene sets from both the RSS and ISS platforms were combined, we observed an improved area under the curve (AUC) value compared to using datasets from either platform alone (Additional file 1: Fig. S4F). Based on these findings, we next compared the top candidate regulators (*P* < 0.05) identified in the RSS and ISS screens to specifically pinpoint senescence regulators involved in inflammaging. A *k*-means clustering of the beta scores demonstrated the existence of two groups of senescence regulators: inflammaging genes and common-aging genes (“Methods”; Fig. 3A). While the common-aging genes were enriched in pathways related to cell proliferation and adipogenesis, the inflammaging genes were involved in novel biological pathways relevant to cellular senescence in inflammaging, such as the complement pathway, the ROS pathway, interferon-gamma response pathway, and the coagulation pathway (Fig. 3B and Additional file 1: Fig. S4G).

Next, we chose to validate four novel candidates accounting for different biological pathways identified from the inflammaging-specific genes, including two genes (*EIF2AK2* and *CMKLR1*) for inflammatory response, one gene (*SERPING1*) for interferon-gamma response, and one gene (*HMGCS2)* for coagulation (Fig. 3C). Inhibiting these four novel inflammaging genes demonstrated the specific pro-senescence effects with IL-6, but not without IL-6 (Fig. 3D–G). Importantly, compared to *TP53*, inhibition of these 4 genes could still allow for the maintenance of MSC identity (Fig. 3H) and normal cell proliferation (Fig. 3I).

**Fig. 4:**
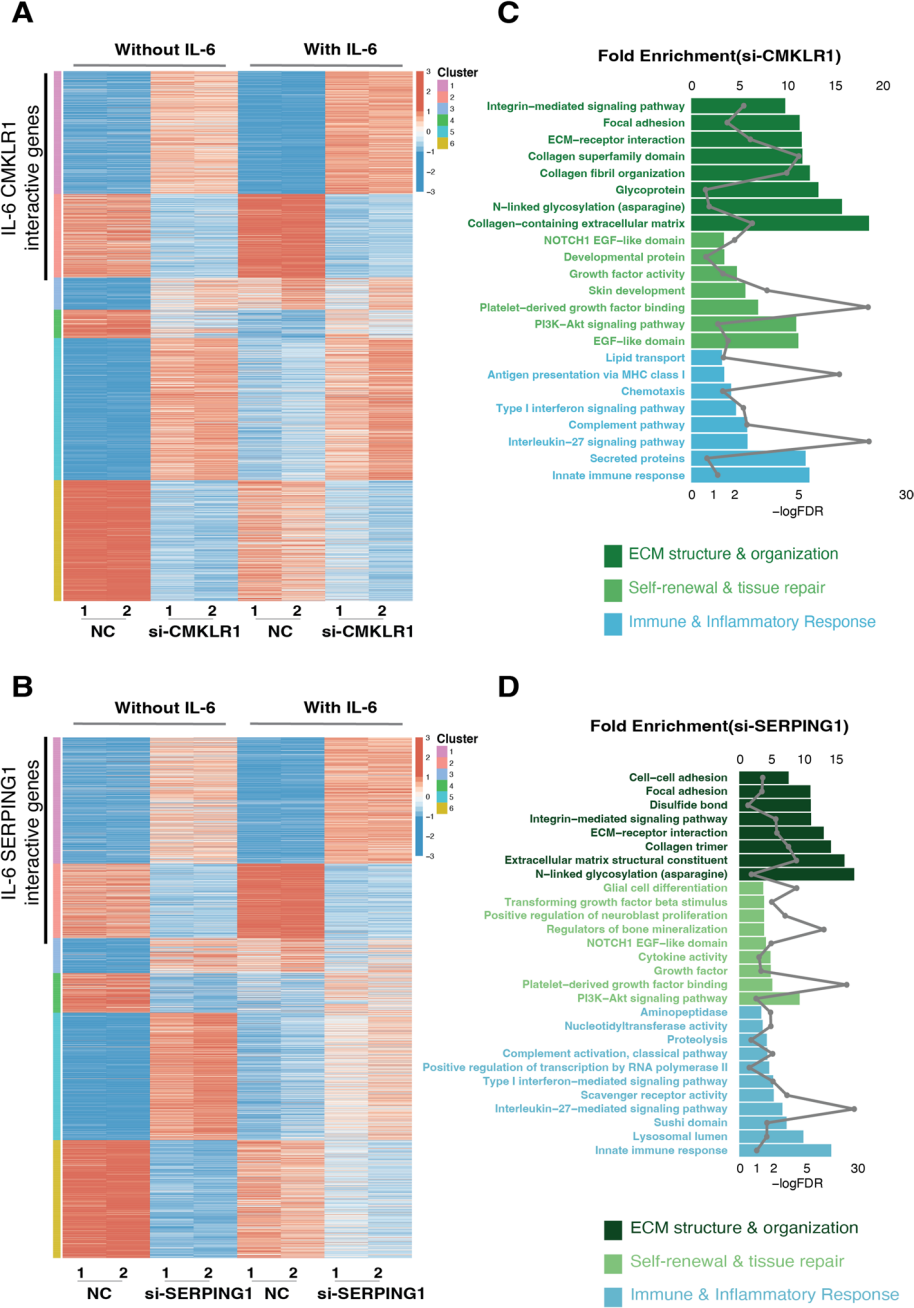
Transcriptome profiles of validated inflammatory-specific senescence genes. **A** *K*-means clustering of DEGs upon inhibition of *CMKLR1* with and without IL-6. **B** Bar plots of the FDR value and fold enrichment for GO term generated from DAVID functional enrichment analysis for Cluster1 and 2 in **A**. **C** *K*-means clustering of DEGs upon inhibition of *SERPING1* with and without IL-6. **D** Bar plots of the FDR value and fold enrichment for GO term generated from DAVID functional enrichment analysis for Cluster1 and 2 in **C**

To interrogate the underlying mechanism for inflammaging, senescence genes, we performed k-means clustering of DEGs upon inhibition of *CMKLR1* or *SERPING1* with and without IL-6. We identified two gene clusters (Cluster1: IL-6 downregulated genes; Cluster2: IL-6 upregulated genes), which function to promote inflammaging senescence but are reversed upon inhibition of either *CMKLR1* or *SERPING1* (Additional file 1: Fig. S5A and B; Fig. 4A and B). Accordingly, we named these two clusters as IL-6_*CMKLR1* interactive genes and IL-6_*SERPING1* interactive genes. Notably, both IL-6_*CMKLR1* interactive genes and IL-6_*SERPING1* interactive genes are significantly enriched in ECM organization, stemness, and immune-modulatory pathways, including type I interferon and IL-27 signaling pathways (Fig. 4C and D). IL-27 is known to counteract IL-6 driven inflammation, and type I IFNs promote anti-inflammatory responses via IL-27 and IL-10 upregulation [36]. Notably, enhanced type I IFN responses and reduced IL-6 levels are associated with healthier aging, especially in younger females [37]. These findings suggest that inhibiting inflammaging-specific senescence genes, *CMKLR1* or *SERPING1*, may reverse the IL-6 associated pro-inflammatory environment during the aging process and rejuvenate the cellular senescence.

In summary, these results demonstrated the usefulness for identifying inflammaging specific genes by comparing the ISS and RSS systems side-by-side, which aids the discovery of new pro-senescence genes relevant to physical or pathological conditions.

### Inflammaging genes contribute to aging pathologies in diverse human organs

**Fig. 5:**
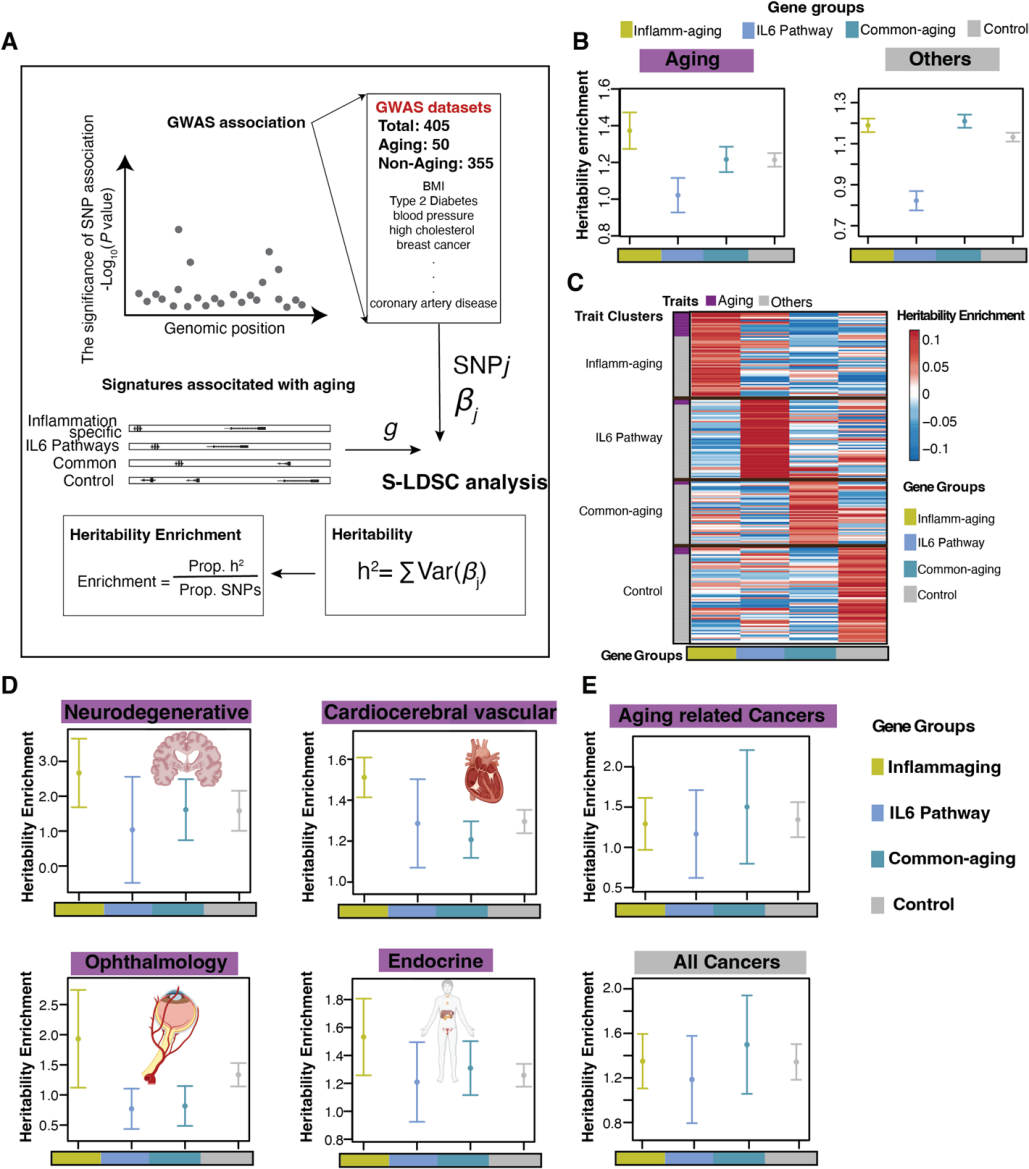
Heritability enrichment analysis of inflammaging genes. **A** The workflow of the S-LDSC analysis. **B** The heritability enrichment estimated by the S-LDSC analysis across aging (left) and non-aging traits (right) for four gene groups: inflammaging, IL-6 pathway, common-aging, and control groups. Each point denotes an estimate, and each error bar denotes ± 2SE. **C** The heatmap of clustered heritability enrichment scores for four gene groups: inflammaging, IL-6 pathway, common-aging, and control groups. At least one of the four gene sets shows heritability enrichment in these traits (aging: purple; non-aging: gray). **D**–**E** The heritability enrichment estimated by the S-LDSC analysis across traits in aging pathology from different organ systems (**D**: neurodegenerative, cardiocerebral vascular, ophthalmology, and endocrine) and in cancers (**E**; top: aging-related cancer traits; bottom: all cancer traits) for four gene groups: inflammaging, IL-6 pathway, common-aging, and control groups. Each point denotes an estimate, and each error bar denotes ± 2SE

To determine how the inflammaging specific regulators identified in our primary MSC cellular senescence model contribute to the organismal aging process, we used the recently published GWAS meta-analysis results to assess their genetic effects in aging traits [38]. Specifically, we collected and curated 405 European-ancestry GWAS datasets, which were further categorized as aging (50) or non-aging (355) studies (Additional file 5: Table S4). Stratified LD score regression (S-LDSC) analysis [39] was performed to calculate the enrichment of common single nucleotide polymorphism (SNP) heritability on each GWAS for four gene sets (inflammaging genes, known IL-6 signaling pathway genes, common-aging genes, and control group genes) (Fig. 5A, Additional file 5: Table S4, Methods). The known IL-6 regulatory pathway genes, which are the genes upregulated by IL-6 via STAT3 [40], were included as a biological control group in this analysis.

Overall, inflammaging genes showed strong enrichment in the common SNP heritability of aging traits (1.37-fold, one-sided *P* = 9.6e − 10). The heritability enrichment for inflammaging genes in aging traits was also significantly higher than that observed for the other three gene groups (left panel in Fig. 5B; inflammaging vs. IL-6 pathway: one-sided *P* = 1.4e − 7; inflammaging vs. common-aging: one-sided *P* = 4.8e − 3; inflammaging vs. control: one-sided *P* = 1.3e − 3). In contrast, inflammaging genes were not enriched in heritability for non-aging traits (right panel in Fig. 5B; one-sided *P* = 0.17). Next, we selected traits for clustering that showed heritability enrichment by at least one of the four gene sets. The analysis identified four distinct types of traits: inflammaging specific traits, IL-6 pathway specific traits, common-aging specific traits, and control traits (top to bottom sorted in Fig. 5C). Inflammaging specific traits are defined as those where inflammaging genes contribute more heritability than the other gene sets. Interestingly, compared with other clusters, the inflammaging specific traits are significantly associated with aging traits (Fig. 5C and Additional file 1: Fig. S6A).

To determine the function of inflammaging genes in aging pathology, we defined aging traits into organ system-based groups (Additional file 5: Table S4) and performed meta-analyses of heritability enrichment across these groups (Methods). Inflammaging genes showed strong enrichment in the common SNP heritability underlying aging pathologies across multiple organ systems, including endocrine (1.53-fold, one-sided *P* = 3.0e − 4), cardiocerebrovascular (1.51-fold, one-sided *P* = 4.5e − 8), ophthalmologic (1.93-fold, one-sided *P* = 9.3e − 3), and neurodegenerative systems (2.67-fold, one-sided *P* = 2.3e − 4). These enrichments were consistently higher than those observed for the other three gene sets across the same aging pathology groups (Fig. 5D; Additional file 1: Fig. S6B).

Notably, inflammaging genes were not enriched for heritability associated with aging-related cancers or other cancer types (Fig. 5E; one-sided *P* ≥ 0.19). These findings are consistent with our experimental validation, suggesting that inflammaging genes contribute functionally to aging pathologies across diverse organ systems, but are not major drivers of tumorigenesis.

## Discussion

A comprehensive and detailed understanding of the molecular basis of natural aging and inflammaging is essential for developing strategies for delaying cellular senescence and aging in humans. Unlike conventional fitness screens that use negative selection to identify essential genes, our study employed positive selection, aiming to detect cells that persist longer under chronic, growth-limiting conditions due to perturbations that delay senescence or promote longevity. Genome-wide CRISPR-based knockout screens have been used to systematically identify potential therapeutic targets for accelerated aging diseases, such as Werner syndrome and Hutchinson-Gilford progeria syndrome. Our work differs fundamentally from these studies. Rather than screening for genes that functionally interact with pathogenic mutations in progeroid syndromes, we performed a genome-wide CRISPRi screen to unbiasedly identify senescence regulators involved in natural aging. We used primary MSCs derived from healthy individuals without pre-existing pathological mutations. To better recapitulate the chronic inflammation in natural aging, we used the inflammatory factor IL-6 to simulate the inflammaging environment in human primary MSCs. Importantly, the CRISPRi system enables loss-of-function perturbation without inducing DNA double-strand breaks (DSBs), thereby avoiding TP53-dependent toxicity associated with DSBs that could confound screening using gene knockout [41, 42].

*TP53*, *CDKN2A (p16*), and *CDKN2D* (*p19*) are well-characterized markers for cellular senescence [43]. However, these genes also play important roles in tumor suppression. Due to their dual functions, recent studies have shown that while enhancing tumor suppression through pathways like *TP53* can protect against cancer, it may also exacerbate certain aspects of aging [44–46]. Thus, targeting tumor suppressors, such as *TP53*, *CDKN2A*, and *CDKN2D*, for therapeutic purposes requires careful balancing between promoting tumor resistance and maintaining long-term cellular function. In contrast, identifying aging markers that operate independently of tumor suppression pathways could open new avenues for developing safer therapeutic strategies to ameliorate aging. Our GWAS meta-analysis showed that inflammaging genes contributed functionally to the aging process but are not associated with tumorigenesis. Consistently, inhibition of four selected inflammaging genes did not impact normal cell proliferation or alter MSC identity. Together, these results suggested that inflammaging genes are novel aging markers independent of tumor suppressing.

Canonical aging pathways identified in model organisms and linked to age-related diseases are nutrient-sensing signaling, translation, proteostasis, stress responses, and genome maintenance. Here, we identified novel gene pathways specific to our ISS system. Particularly, *SERPING1*, an inhibitor of complement component 1 (C1), was further validated as a regulator that alleviates MSC senescence in inflammaging (Fig. 3D–G, Fig. 4B and D). Transcriptome analysis further demonstrated that *SERPING1* perturbation induces an anti-inflammatory and rejuvenated transcriptomic state in MSC (Fig. 4B and D).

Interestingly, *SERPING1* knockdown also showed enrichment in pathways related to lysosomal function, suggesting a role in autophagy-related processes. Autophagy, a lysosome-mediated degradation mechanism [47], is essential for intracellular turnover and supports MSC differentiation and maintenance [48] which may promote a youthful and regenerative MSC phenotype. Thus, *SERPING1* knockdown likely enhances MSC regenerative potential through improved autophagic activity and subsequent ECM restructuring. These mechanistic clues warrant further studies, representing a promising avenue of investigation. Moreover, our results are consistent with previous evidence that genetic variation in *SERPING1* significantly alters susceptibility to age-related macular degeneration [49]. With advancing age, an increasing number of healthy individuals have laboratory signs of increased coagulation enzyme activity [50]. Our study is the first to prove coagulation genes could regulate the MSC senescence, creating a link between coagulation enzymes and cellular senescence and aging [51]. Furthermore, inhibition of *SERPING1* did not affect the normal function of cell proliferation or alter MSC identity (Fig. 3H–I), suggesting that *SERPING1* presents a tumor suppressor-independent marker for ameliorating inflammaging.

MSCs have generated great interests in regenerative medicine and immunotherapy due to their unique biological properties, such as self-renewal, multipotency, and immunosuppression. However, human MSC batches vary significantly in their functional characteristics, impacting the effectiveness for therapies. The effectiveness of MSC-based therapies may be enhanced by defining determinant molecular biomarkers to predict MSC function. To address this, researchers have developed the Clinical Indication Prediction (CLIP) scale to predict the therapeutic efficacy of a given MSC donor population based on *TWIST1* expression levels [52]. However, the heterogeneity of MSC requires more comprehensive biomarkers to predict the fitness across different MSC batches. The pro-aging signatures identified from our ISS system provide a rich set of markers for future evaluation. Notably, we provided numerous potential targets for engineering and optimizing MSC cell therapy products without changing the properties of MSC.

Our study, along with others, has demonstrated that functional genomics approaches, including CRISPRi [53–55], CRISPRa screens [56, 57], and genomic regulatory network analyses [38, 58–60] can improve the identification of causal variants and improve heritability estimation for non-coding SNPs. We integrated inflammaging genes identified from our CRISPRi functional screens to estimate heritability in aging traits and demonstrated that common SNPs within 100 kb distance to the identified inflammaging genes contribute to the heritability of aging pathologies across diverse human organs. Notably, inflammaging genes were strongly enriched in the common SNP heritability underlying neurodegenerative diseases (Fig. 5D), including Alzheimer’s disease (AD) (2.67-fold, one-sided *P* = 2.3e − 4, Additional file 5: Table S4). Previous studies have established that aging is the most important risk factor for AD [61]. Our finding further pinpoints that inflammaging genes are critical components in the pathogenesis of AD [62], which may serve as potential therapeutic targets for AD.

## Conclusions

Together, our findings offer a comprehensive resource of aging regulators to guide future research on therapeutic targets, supporting the development of aging interventions and more effective cellular therapies for aging-related diseases, including cancer.

## Methods

### Study design

The goals of this study were (i) to identify novel senescence-promoting genes in adult stem cells, (ii) to characterize novel senescence-promoting genes in a specific inflammatory milieu, and (iii) to decipher the inflammatory aging process by comparing two solid screening platforms, thus highlighting the biomarker for pathological aging and therapeutic potential of MSC by manipulating the senescence-promoting genes. In vitro assays such as the SA-β staining and proliferation rates of primary MSC were carried out at least three independent times. Sample sizes were calculated by the investigators based on previous experience. Images from colorimetric SA-β-gal  staining were analyzed with the investigator blinded to the treatment received.

### Cell culture

Primary adipose derived cells (Lifeline) were maintained on gelatin-coated tissue culture plates with medium specific for MSC growing (StemLife MSC Medium Complete Kit, Lifeline). Human embryonic kidney (HEK293T) cells (ATCC) were cultured in 10% fetal bovine serum (Thermo Fisher Scientific) in DMEM (Thermo Fisher Scientific). Cells were maintained at 37 ℃ in a cell incubator containing 5% CO_2_. Cell authentication and mycoplasma testing were performed either by the vendor (as documented in the certificate of analysis) or confirmed in-house prior to large-scale experimental use.

### Plasmids construction

To clone individual sgRNAs targeting different candidate genes from the screening experiments, the lentiviral vector (pSLQ1373) expressing an optimized sgRNA was linearized and gel purified. New sgRNA sequences were PCR amplified from pSLQ1373 using different forward primers and a common reverse primer, gel purified, and ligated to the linearized pSLQ1373 vector using In-Fusion cloning (Clontech). Primers used to construct individual sgRNAs are shown in Additional file 6: Table S5.

### Lentivirus production

HEK293T cells were seeded at ∼30% confluence 24 h before transfection. Lentivirus was produced by co-transfecting with pHR plasmids and encoding packaging protein vectors (pMD2.G 165 ng and pCMV-dR8.91 1.32ug) using TranslT-LT1 transfection reagents (Mirus, 6-well plate, could be scaled up). Viral supernatants were collected 72 h after transfection and filtered through a 0.45 μm strainer (Millipore). For concentrating lentivirus, 1 volume of lentivirus supernatant was mixed with 4 volumes of lentivirus precipitation solution (Alstem) and was reserved at 4 °C overnight. Then the concentrated lentivirus was pelleted at 1500 × g for 30 min at 4 °C. Supernatant was used for transduction immediately or kept at − 80 °C for long-term storage.

### High-throughput screening for senescence of MSC

hCRISPRi-v2 library was a gift from Jonathan Weissman [16, 18] (Addgene ID #83,969; http://n2t.net/addgene:83969; RRID:Addgene_83969).

The senescence screens were performed in two independent replicates. For each replicate, a parallel experiment was conducted for the RSS and ISS platforms.

1. On day − 3, 125 M MSC cells were seeded at 2.5 M cells/15 cm gelatin-coated dishes.
2. On day − 1, cells were transduced with a pooled lentiviral sgRNA library with a MOI of 0.3.
3. On day 0, cells were switched to basal medium with and puromycin at 2 μg/mL.
4. Seven days after puromycin selection, 25 million cells were harvested as the initial population of the screen.
5. Fresh medium with puromycin at 1 μg/mL was changed every day starting day 7.
6. On day 14, cells integrated lentiviral sgRNA library were transduced with a lentiviral construct pSLQ6604 that expresses dCas9-KRAB from SFFV promoter.
7. On day 17, cells were harvested and conducted a two-part parallel experiment: one experiment is for growing naturally until their growing limitation, which is as the RSS platform; one experiment is for growing in the presence of 10 ng/ml recombinant human IL-6 protein (Abcam, ab259381) until their growing limitation, which is as the ISS platform. Fresh medium was changed every 2 days.
8. About 7–8 weeks later, cells were harvested, and genomic DNA was extracted from all samples: the sgRNA-encoding regions were then amplified by PCR using HiSeq forward and reverse primers and then sequenced on an Illumina HiSeq-4000 using HiSeq custom primer with previously described protocols at high coverage. Primers used are summarized in Additional file 6: Table S5.

### RNA extraction and quantitative RT-PCR

Cells were lysed by using Accutase (STEMCELL), and total RNA was extracted by using the RNeasy Plus Mini Kit (QIAGEN). By using iScript cDNA Synthesis Kit (Bio-Rad), reverse transcription was performed. Quantitative PCR reactions were prepared with iTaq Universal SYBR Green Supermix (Bio-Rad) and were run on a LightCycler thermal cycler (Bio-Rad). Primers used are summarized in Additional file 6: Table S5. All experiments were performed according to the manufacturer’s instructions.

### RNA-seq and analysis

Bulk RNA-seq was performed by Novogene using the Illumina NovaSeq PE150 platform (150 bp paired-end). Raw reads were trimmed to remove adaptors and assessed for quality using FastQC. The QC matrix of RNA-seq was listed as Additional file 7: Table S6. High-quality reads were aligned to the human reference genome (GRCh38.p13) using HISAT2, and transcript assembly was performed with StringTie. Differential expression analysis was conducted using DESeq2. Genes with fold change ≥ 1.5 or ≤ 0.67 and *P* < 0.05 were considered significantly upregulated or downregulated, respectively. Pathway enrichment analysis was conducted to identify significantly pathways by online gene functional annotation tools (https://davidbioinformatics.nih.gov/).

### Flow cytometry

Primary MSCs with different verified sgRNAs were harvested, washed with ice-cold PBS containing 2% FBS, and adjusted to a concentration of 10^7^ cells/mL. Cells were stained and incubated with diluted antibodies (listed in Additional file 6: Table S5) as 5 µl per million cells in 100 µl staining volume at 4 °C for 30 min in Eppendorf tubes. After staining, cells were washed two times by centrifugation at 400 g for 5 min and resuspended in 100 μL ice-cold PBS. Cells were kept in the dark on ice and analyzed for fluorescence using a CytoFLEX S flow cytometer (Beckman Coulter). Data were analyzed using FlowJo v10.8.1 (BD Biosciences).

### SA-β-gal staining

Two methods were used to quantify the analysis of SA-β-gal positive cells.

By flow cytometry: MSC with different modified genes were seeded, respectively, in a 6-well plate and cultured overnight in a 5% CO_2_ incubator. The cells were washed with 2 ml of PBS once. Bafilomycin A1 working solution (1 ml) was added to the culture dish, and the cells were incubated for 1 hour in a 5% CO_2_ incubator. SPiDER-βGal working solution (1 ml) was then added to the culture dish, and the cells were incubated for 30 min in a 5% CO_2_ incubator.After the supernatant was removed, the cells were washed with 2 ml of PBS twice. The cells were harvested by trypsin and resuspended in PBS containing 2% FBS and were analyzed by a flow cytometer (Beckman Coulter) (excitation: 488 nm, emission: 515–545 nm).

By microscope: MSC cultured in 12 well-plates were washed once with 1 mL of 1XPBS and fixed in 0.5 mL of fixative solution for 10–15 min at room temperature. After washing fixed MSC twice with 1XPBS, 0.5 mL of the Staining Solution Mix were added to each well, and the cells were incubated at 37 °C for 6 h in a Ziplock® resealable bag to avoid any effect from the CO_2_. Then, the cells were observed under a microscope for the development of blue color (200× total magnification). Images were taken, and the percentages of positive cells were calculated and analyzed using ImageJ.

### Cell proliferation detection

Briefly, MSCs were labelled by CellTrace™ (Invitrogen) as the manufacturer’s indication. Stock solution was diluted 1000-fold immediately prior to use. Grow the MSCs with different sgRNAs to approximately 0.1 M per 10 cm dish.Dilute the CellTrace™ stock solution in pre-warmed (37 °C) phosphate-buffered saline (PBS) to the working concentration (10 μM). Remove the culture medium from the cells and replace it with the loading solution. Incubate the cells for 20 min at 37 °C. Remove the loading solution, wash the cells twice with culture medium, and replace it with fresh, pre-warmed complete culture medium. Incubate the cells with medium containing different concentrations of IL6 for 3 days. Analyze the stained cells with a CytoFLEX S flow cytometer (Beckman Coulter). Data were analyzed using FlowJo v10.8.1 (BD Biosciences) with a plugin for proliferation index.

Other methods to monitor cell proliferation were by using cell counting kit 8 (Abcam, ab228554). Briefly, plate 5000 MSCs of different numbers of passages per well in a tissue culture microplate and incubate them in a 37 °C, 5% CO_2_ incubator for 72 h. Add 10 μl/well of WST-8 Solution to each well. Protect from the light and incubate for 2 h at 37 °C. Measure the absorbance increasing at 460 nm.

### Sequence alignment, the calculation of enrichment score, quality control

We calculated the gRNA count matrix by extract match for each read (Additional file 2: Table S1). The gRNA count table was loaded into MaGeCK (Model-based Analysis of Genome-wide CRISPR-Cas9 Knockout) [63] to use the maximum likelihood estimation (MLE) approach to estimate the essentiality of genes in cell senescence by comparing the experimental in replicative arrest and inflammation (IL6 stimulation) condition. The beta score was calculated to call gene essentialities: a positive beta score means a gene is positively selected (the gene expression positively functions in cell senescence), and a negative beta score means a gene is negatively selected (the essential genes or the gene expression negatively functions in cell senescence). Quality control was performed across multiple aspects, such as the sequence quality level, read count level, sample level. The QC matrix from MaGeCK was shown as Additional file 2: Table S1. The false discovery rate (FDR) for each gene was obtained by performing 100 permutations of the read count data to generate the null distribution. In each round, the sgRNA assignments were randomly shuffled, and null *P*-values for each gene were generated using MaGeCK. These null P-values were then used to estimate the FDR for each gene. The results are provided in Additional file 2: Table S1.

To further get high level of quality control, ROC indicators were calculated by using predefined essential genes (KEGG_Ribosome, PMID: 33010154) and non-essential genes (PMID: 27111720) as positive and negative controls. Besides, a curated pro-senescence gene list defined as genes upregulated during senescence in at least two out of eight senescence-associated public databases and predefined essential genes (KEGG_Ribosome, PMID: 33010154) as positive selection and negative selection. We generated ROC curves and calculated the area under the curve (AUC) values using beta scores and *P*-values from MaGeCK for RSS, ISS, and combined RSS + ISS datasets via logistic regression models. To calculate the *P*-value for the AUC, we performed 100 permutations of the read count data to generate its null distribution. All the results yield *P*-value < 0.01.

### The definition of inflammaging gene set from *K*-means clustering

The beta score of signature genes (*P*-value < 0.05) from RSS screening or ISS screening was used to perform the unsupervised clusters (*K*-means) and plot the heatmap (Fig. 3A). Based on the clustering result, the beta scores from the MaGeCK MLE approach were used as input in TreeView to plot the heatmap figures. The genes in the first two clusters are specifically enriched in ISS screening, not RSS screening, thus defined as “inflamm-aging gene”. The genes in the third and fourth clusters are consistently enriched in ISS screening and RSS screening, thus defined as “common-aging gene”. Genes included in each hallmark gene set were based on MSigDB (Molecular signature database) [64, 65]. The P-value was calculated by hypergeometric test.

### Pathway enrichment analysis

Pathway enrichment was performed using Metascape [66] (https://metascape.org), which integrates multiple gene set libraries, including MSigDB (Canonical and Hallmark), KEGG, and GO Biological Processes. Enrichment significance was calculated using the cumulative hypergeometric distribution, with multiple testing corrected by the Benjamini–Hochberg method (*q*-value). As the gene list was derived from an unbiased genome-wide CRISPRi screen, the default background—the complete human proteome—was used. Pathways were prioritized by enrichment factor and *p*-value (< 0.01) and considered significant at *q*-value < 0.25 (Additional file 4: Table S3), a threshold for exploratory studies to balance sensitivity and specificity.

### GWAS data

We collected 405 European-ancestry GWAS datasets, including 50 aging-related and 355 non-aging studies (Additional file 5: Table S4). The sample size of these datasets ranged from 14,267 to 1,320,016, with a median of 452,264. All datasets had observed-scale heritability *Z*-score ≥ 6 as estimated by S-LDSC (see below). All datasets were processed as previously described [39], and they were annotated as aging-associated traits or non-aging-associated traits. The traits associated with aging pathologies were further grouped by different organ systems (Additional file 5: Table S4).

### Gene set heritability enrichment based on GWAS

We defined four gene sets: inflamm-aging genes, known IL6 signaling pathway genes, common-aging genes, and control group genes (Additional file 5: Table S4). The inflamm-aging genes and the common-aging genes are defined by Fig. 3A. While the common-aging genes were enriched in both RSS and ISS screens, the inflammaging genes specifically promoted cellular senescence in inflammaging. The known IL6 signaling pathway genes are 87 genes in the gene list named as “HALLMARK_IL6_JAK_STAT3_SIGNALING” in Molecular Signatures Database (MSigDB) [40, 64], which was used as a biological control pathway in this analysis. The control group genes are randomly selected genes from the whole genome. Additionally, these genes exclude the genes in the two gene groups: the inflamm-aging genes and the common-aging genes.

We used a previously developed pipeline [38] to assess heritability enrichments of gene sets. For each of the 4 gene sets, we first created the corresponding binary SNP annotation using a distance-based approach from previous studies [67, 68]. Specifically, we annotated each SNP as being “inside” a gene set if it is within 100 kb of the transcribed region of a gene in the gene set. Next, we integrated each SNP annotation with the summary statistics of each GWAS using S-LDSC [39] (version 1.0.1, https://github.com/bulik/ldsc) conditional on 96 annotations from the baseline-LD model [68] (version 2.2, https://alkesgroup.broadinstitute.org/LDSCORE). The 96 baseline-LD annotations capture diverse functions in the genome such as translation, regulation, selection and conservation. For each SNP annotation based on a given gene set, S-LDSC estimates the heritability enrichment as $\left[{h}_{a}^{2}/{h}^{2}\right]/\left[\left|a\right|/p\right]$, where $\left|a\right|$ is the number of common SNPs (MAF ≥ 0.05), $p=$ 5,961,159 is the total number of common SNPs, and ${h}_{a}^{2}$ and ${h}^{2}$ are heritabilities due to $|a|$ common SNPs with annotation $a$ and $p$ common SNPs respectively. Because the 405 GWAS datasets were obtained from populations of European descent, we used haplotypes of individuals with European ancestry from 1000 Genomes Phase 3 [69] to construct the LD reference panel for the S-LDSC analysis of all 4 gene sets. The S-LDSC results of 4 gene sets and 405 GWAS are available in Additional file 5: Table S4.

We meta-analyzed heritability enrichment results across all GWAS datasets in the same trait group (Fig. 4B, D) as previously described [39]. Specifically, we performed random-effects meta-analyses of individual estimates and standard errors (SEs) from S-LDSC to obtain the meta-analyzed estimate and SE for each trait group, using the function “meta.summaries” from R package rmeta (version 3.0, https://cran.r-project.org/web/packages/rmeta). To find the *P*-value for meta-analyzed heritability enrichment in each trait group, we first meta-analyzed $\left({h}_{a}^{2}/\left|a\right|\right)-\left[\left({h}^{2}-{h}_{a}^{2}\right)/\left(p-\left|a\right|\right)\right]$ and then computed a one-sided *Z*-score to test if this difference is greater than 0.

### Statistical analysis

For quantification of differentiation efficacy by FACS, values represent the average value of three biologically independent experiments. For quantification of gene expression by realtime-PCR, gene expression levels were normalized to the housekeeping gene has-GAPDH. Data are represented as mean from 2 biological replicates, each individually performed in technical triplicates. Error bars represent standard deviations that are calculated in. For SA-β gal staining assays, *n* represents the random number of magnification fields of view recorded by a blinded investigator. Error bars represent standard deviations that are calculated in Prism 9 (GraphPad). Detailed statistics, including methods and p-values, are provided in Additional file 8: Table S7.

## Supplementary Information

Additional file 1: Supplementary Figures 1-6.

Additional file 2: Table S1: The gRNA count matrix and QC matrix for sgRNA library.

Additional file 3: Table S2: The lists of well-characterized pro-senescence gene.

Additional file 4: Table S3: The GO-term enriched by Metascape for Fig. 1C and Fig. 2B.

Additional file 5: Table S4: Heritability enrichments of four gene sets across 405 GWAS datasets.

Additional file 6: Table S5: Primers, sequence of guide sgRNAs and antibody in the study.

Additional file 7: Table S6: QC matrix for RNA-seq.

Additional file 8: Table S7: Statistic test table in the study.

## Data Availability

The CRISPRi screen data and RNA-seq data have been deposited in the Gene Expression Omnibus under the accession ID GSE268569 [70] and GSE300879 [71]. This study did not involve the use of any custom code. All analyses were performed using standard, publicly available software tools as described in the Methods section.
