# Supplementary material for: Identification of replicative aging and inflammatory aging signatures via whole-genome CRISPRi screens
Source: Genome Biol. 2025 Aug 6;26:233. doi: 10.1186/s13059-025-03683-7 (PMC12326738; doi:10.1186/s13059-025-03683-7)
Supplement: Supplementary file 1 — Additional file 1: Supplementary Figures 1-6. [file 13059_2025_3683_MOESM1_ESM.docx]

**Supplemental Figures**

**
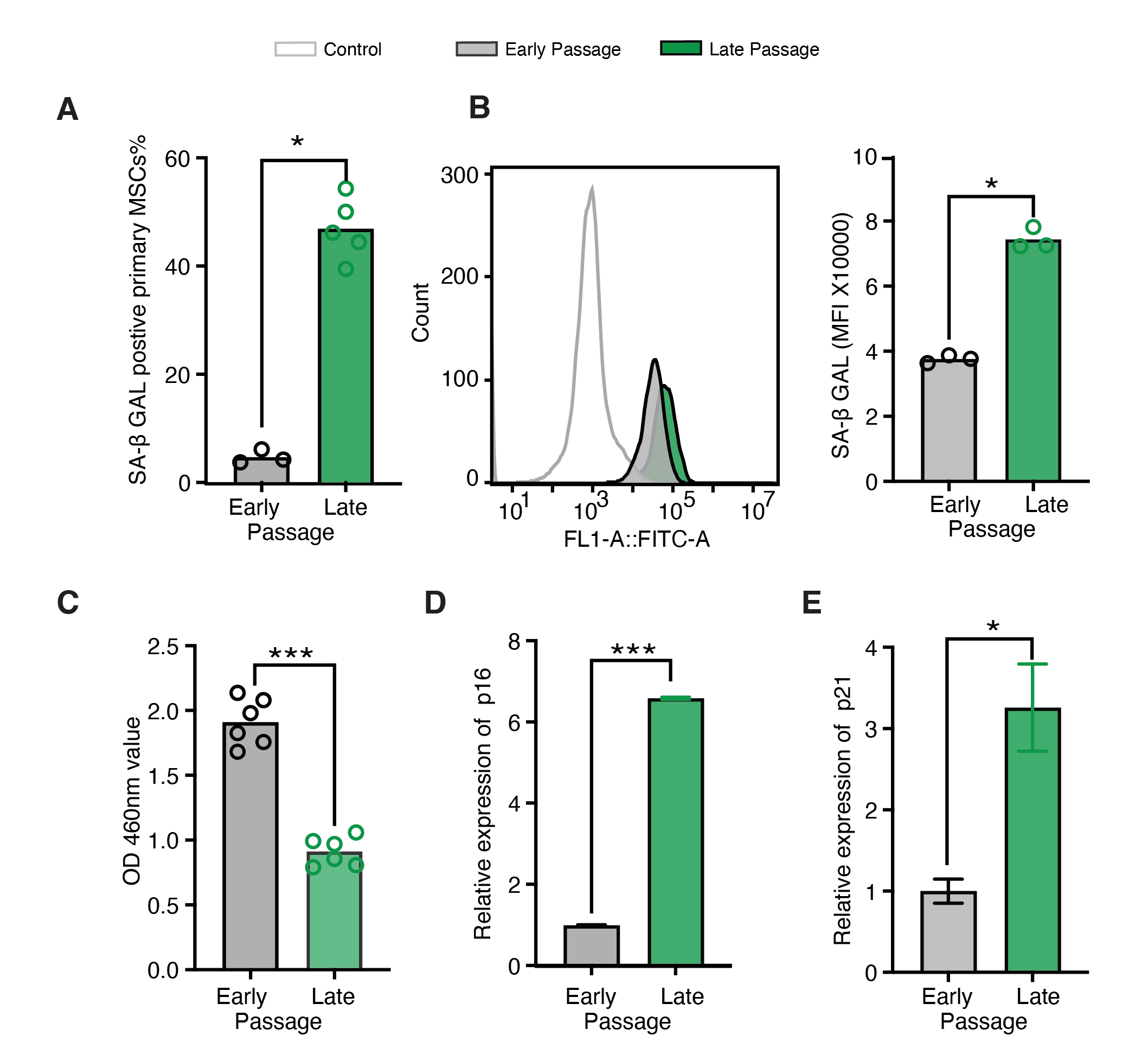
**

**Fig. S1**

(A) Quantification of SA-β-gal staining of human primary MSC with different number of passages by light microscopic. (B) Quantification of SA-β-gal staining of human primary MSC with different number of passages by flowcytometry. n=3 biological replicates. (C) Cell proliferation rates measured by CCK8 methods. n=6 biological replicates. (D) Expression of *p16*, a cellular senescence molecular biomarker, in human primary MSC with different number of passages. (E) Expression of *p21*, a cellular senescence molecular biomarker, in human primary MSC with different number of passages. n=2 biological replicates with 3 technical replicates. (*p < 0.05, **p<0.01, ***p<0.001, ns non-significant)


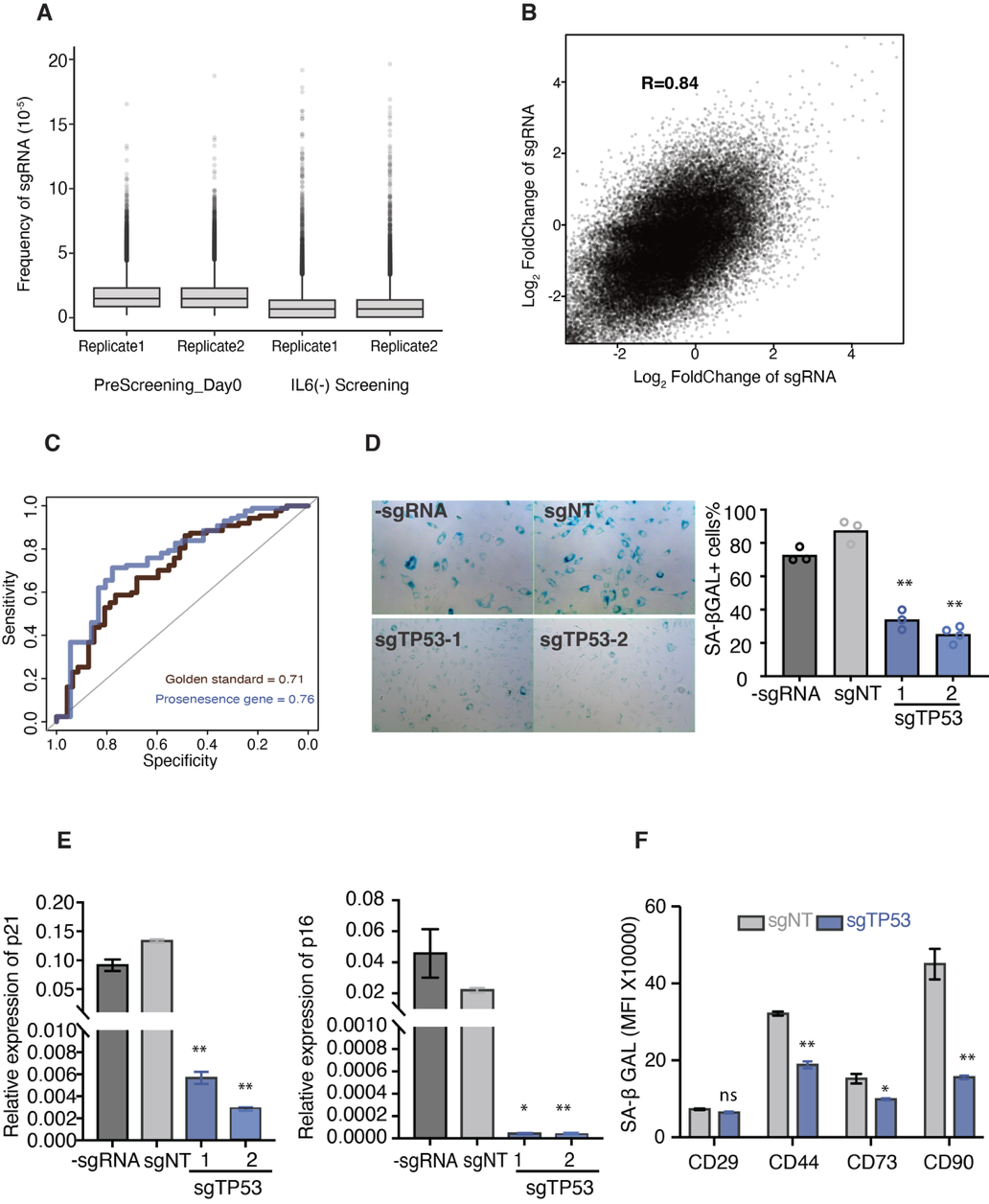


**Fig. S2**

(A) Barplot showing the abundance of all sgRNAs from RSS in two replicate experiments. (B) Scatter plot showing Log_2_ fold change of sgRNA abundance between final passage and initial passage in two replicates. (C) ROC curve comparing the classification performance of RSS platform by 2 different gene-sets. The p values of AUC were calculated based on 100 permutations of the read count data. (p < 0.01). (D) SA-β-gal staining of human primary MSC upon perturbation of TP53. (E)Expression of p16 and p21 in human primary MSC upon perturbation of *TP53*. (F) Expression of four different biomarkers for MSCs upon perturbation of *TP53* (*p < 0.05,**p<0.01,ns non-significant).


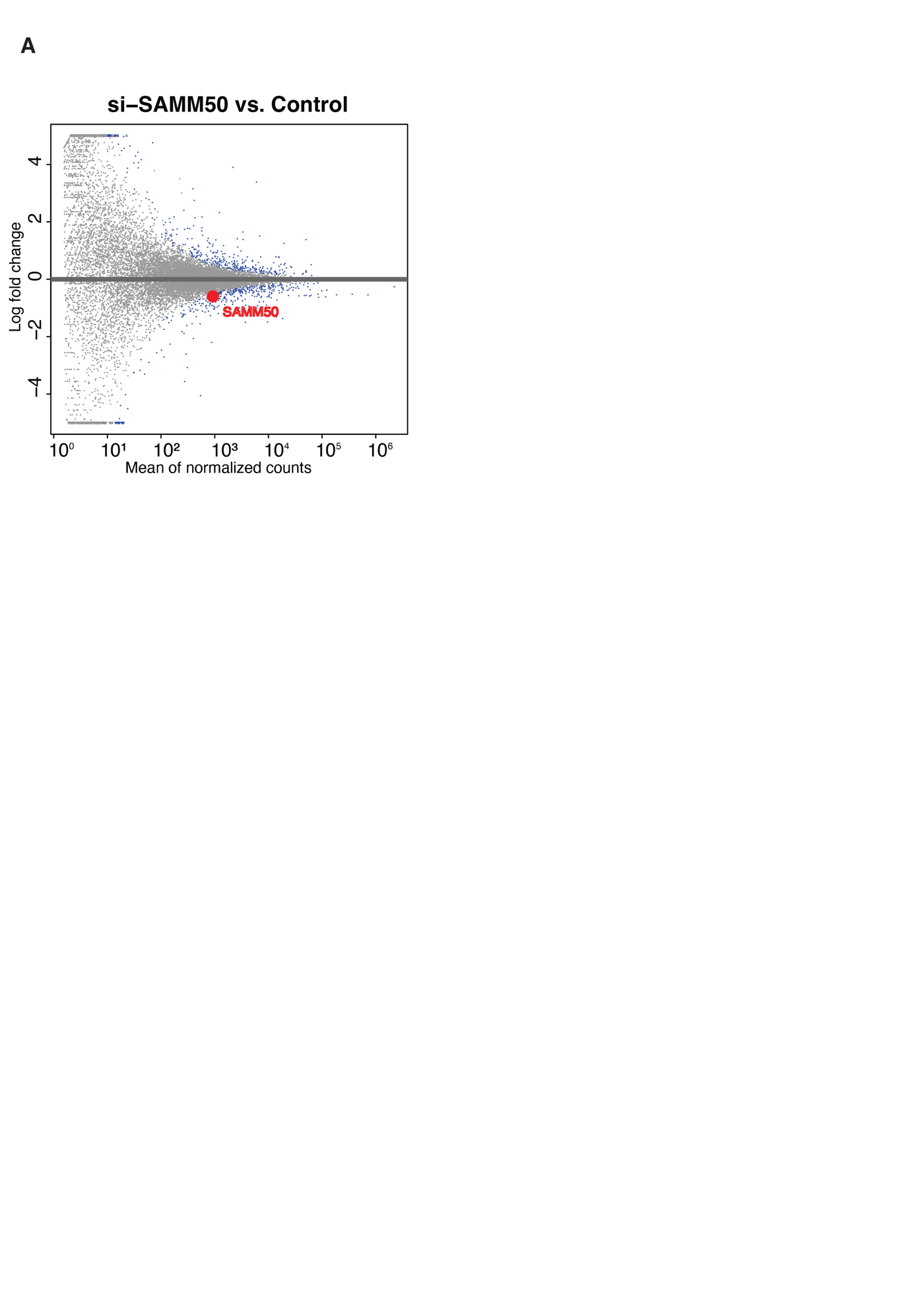


**Fig. S3**

(A) MA plots illustrating gene expression changes upon inhibition of SAMM50 in primary MSCs. The red dot represents the gene SAMM50.

**
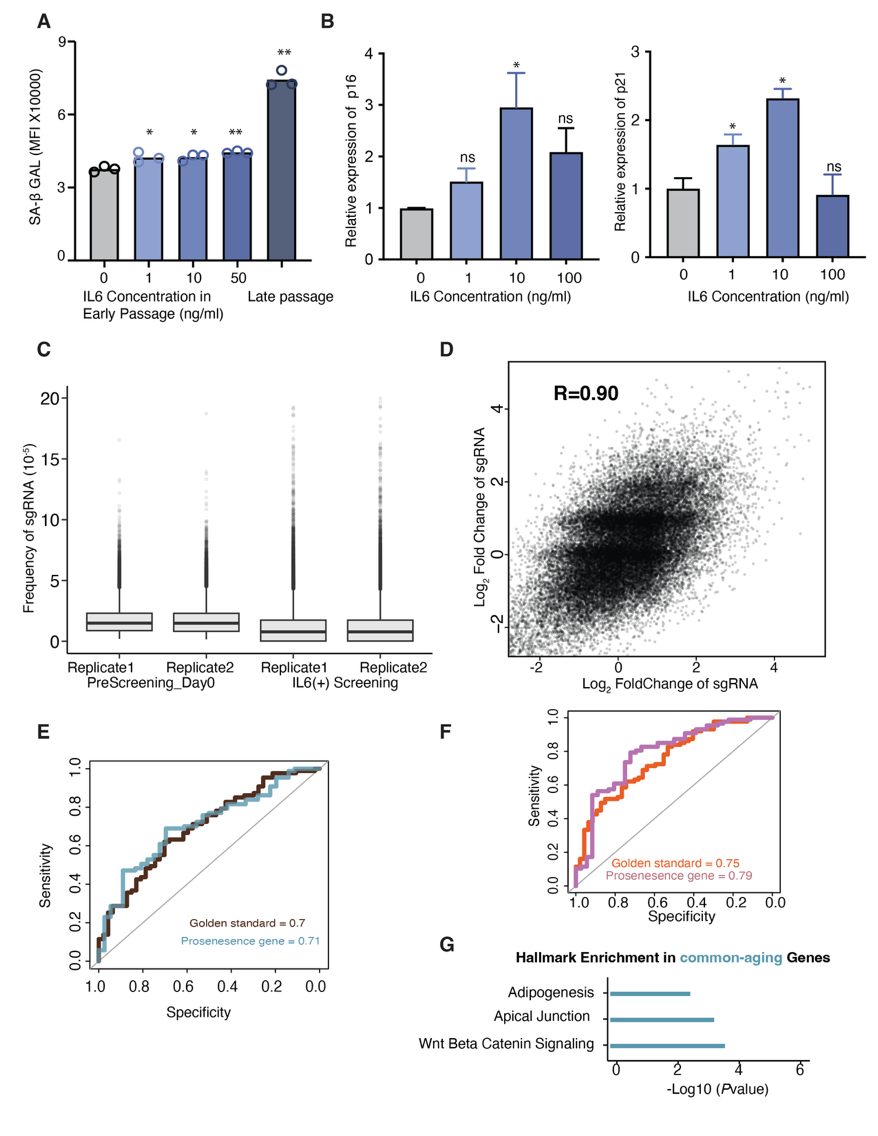
**

**Fig. S4**

(A) SA-β-gal staining of human primary MSC treating with different concentration of IL-6. (B) Expression of p16 and p21 in human primary MSC treating with different concentration of IL-6. (C) Barplot showing the abundance of all sgRNAs from ISS in two replicate experiments. (D) Scatter plot showing Log2 fold change of sgRNA abundance between final passage and initial passage in two replicates. (E) ROC curve comparing the classification performance of ISS platform by 2 different gene-sets. The p values of AUC were calculated based on 100 permutations of the read count data (p < 0.01). (F) ROC curve comparing the classification performance of the combination analysis of RSS and ISS platform by 2 different gene-sets. The p values of AUC were calculated were calculated based on 100 permutations of the read count data (p < 0.01). (G) Representative Hallmark genes enriched in the common-aging genes identified in the Fig. 3A.


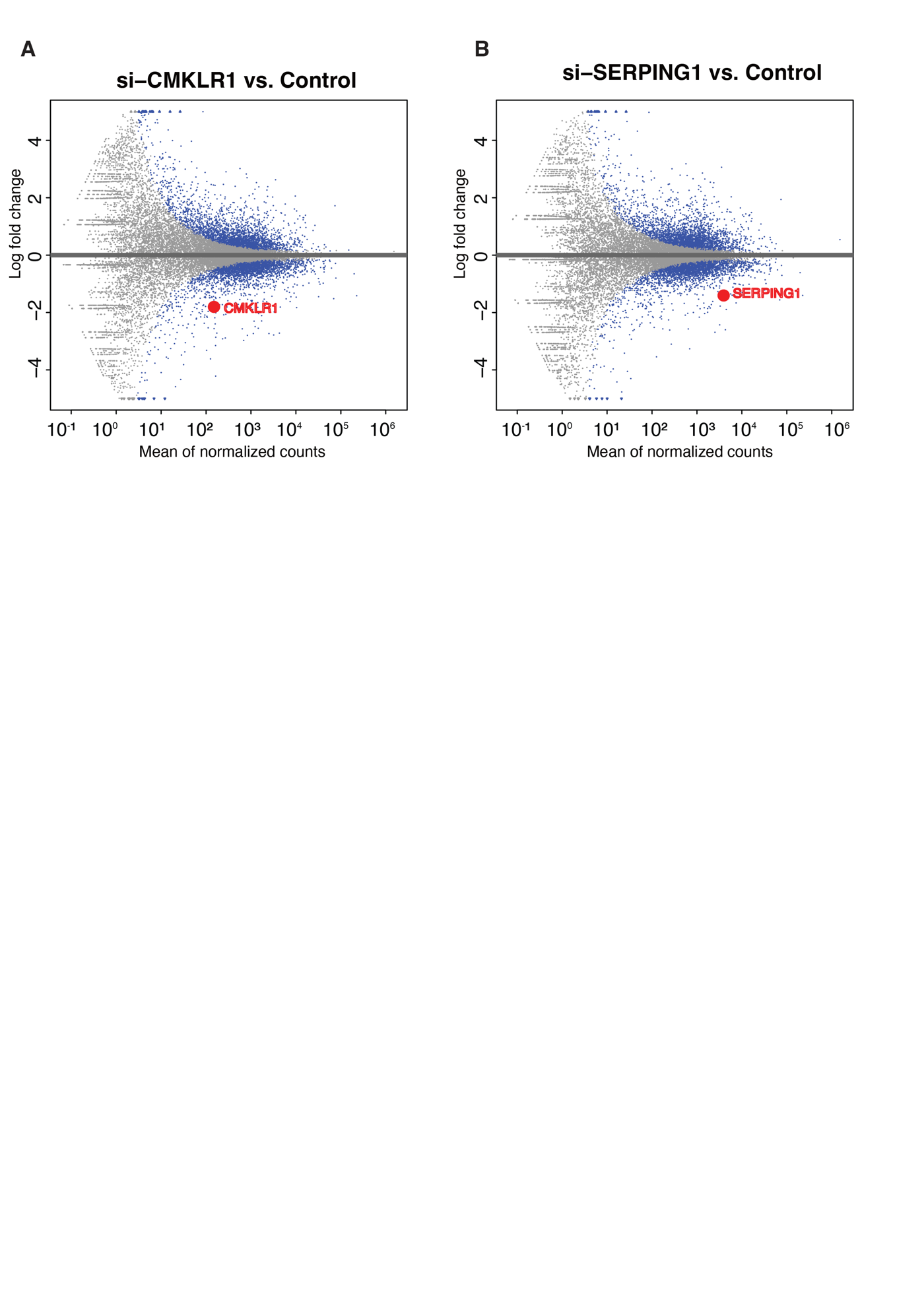


**Fig. S5**

MA plots illustrating gene expression changes upon inhibition of *CMKLR1* (B) and *SERPING* (C) in primary MSCs. The red dot represents the gene *CMKLR1* (B) and *SERPING* (C).

**
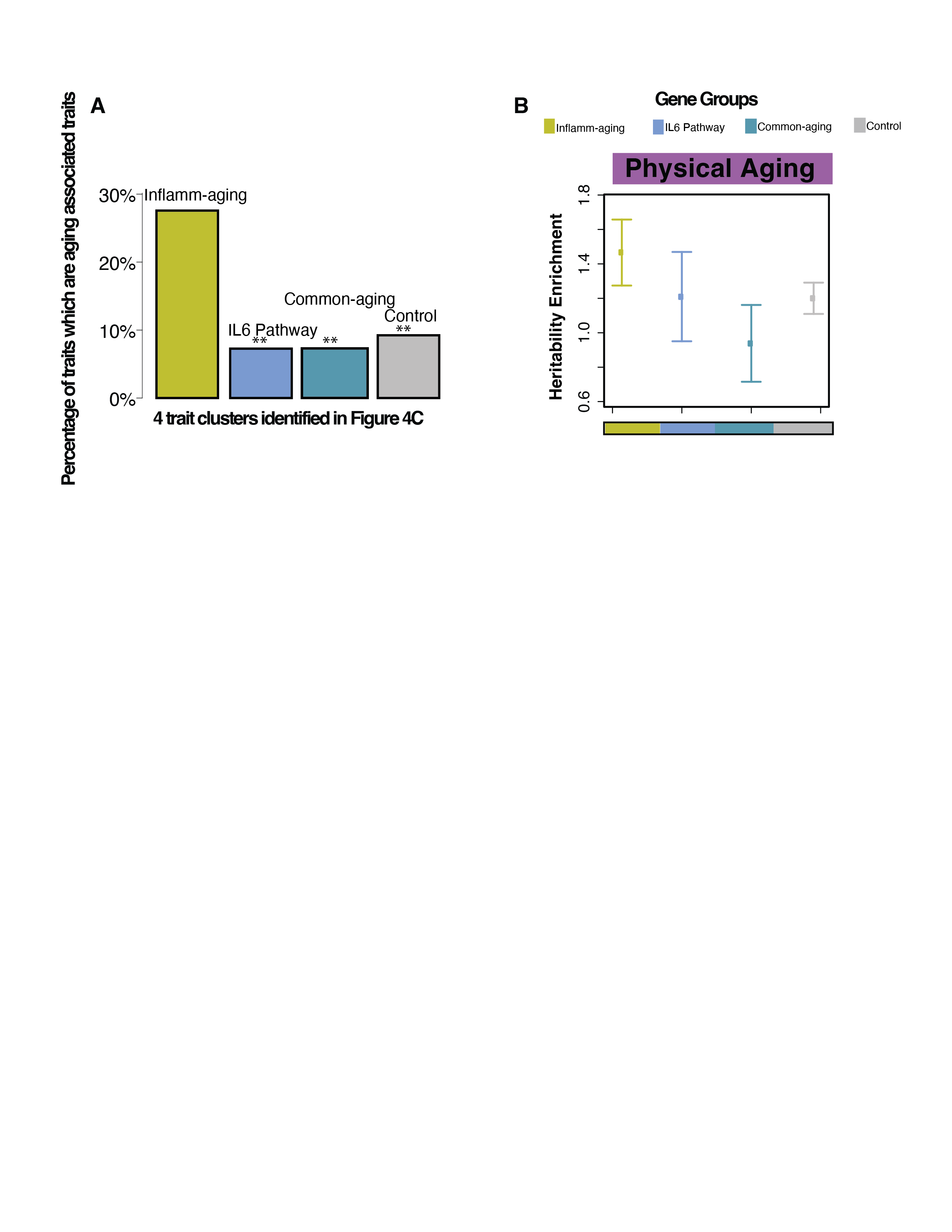
**

**Fig. S6**

(A) The percentage of aging traits in four trait clusters identified in **Fig. 5C**. (*p < 0.05, **p<0.01, one-sided fisher’s test). (B) The heritability enrichment estimated by the S-LDSC analysis across physical aging traits for four gene groups: inflammaging, IL-6 pathway, Common-aging and control groups.
